# Supplementary material for: Kappa opioid receptor antagonism restores phosphorylation, trafficking and behavior induced by a disease-associated dopamine transporter variant
Source: Mol Psychiatry. 2025 May 29;30(10):4651–64. doi: 10.1038/s41380-025-03055-4 (PMC12436197; doi:10.1038/s41380-025-03055-4)
Supplement: Supplementary file 2 — Suppl Table 1 [file 41380_2025_3055_MOESM2_ESM.docx]

**Supplementary Table S1**

| Table S1: statistical analysis | | |
| --- | --- | --- |
| Figure 1A | One-Way ANOVA | F (DFn, DFd): F (7, 16) = 10.02; P<0.0001 |
| Figure 1B | One-Way ANOVA | F (DFn, DFd): "F (7, 32) = 7.957"; P<0.0001 |
| Figure 1 C (vmax) | Unpaired t-test, two-tailed | t, df: t=5.931, df=4; P=0.0040 |
| Figure 1C (KM) | Unpaired t-test, two-tailed | t, df: t=0.6160, df=4; P=0,5712 |
| Figure 1D | One-way ANOVA | F (DFn, DFd): F (5, 28) = 6.280; P=0.0005 |
| Figure 1E | One-way ANOVA | F (DFn, DFd): F (3, 12) = 9.307; P=0.0019 |
| Figure 1F (vmax) | Unpaired t-test, two-tailed | t, df: t=1,723, df=4; P=0.1600 |
| Figure 1F (KM) | Unpaired t-test, two-tailed | t, df: t=1.896, df=4; P=0.1309 |
|  | | |
| Figure 2A (55-60 kDa) | two-way ANOVA | Interaction |
|  |  | F (1, 8) = 0.009013; P=0.9267 |
|  |  | Genotype |
|  |  | F (1, 8) = 0.02481; P=0.8787 |
|  |  | Drug |
|  |  | F (1, 8) = 0.2537; P=0.6280 |
| Figure 2A (85-90 kDa) | two-way ANOVA | Interaction |
|  |  | F (1, 8) = 0.6778; P=0.4342 |
|  |  | Genotype |
|  |  | F (1, 8) = 68.25; P<0.0001 |
|  |  | Drug |
|  |  | F (1, 8) = 0.8756; P=0.3768 |
| Figure 2B (55-60 kDa) | two-way ANOVA | Interaction |
|  |  | F (1, 8) = 5.715; P=0.0438 |
|  |  | Genotype |
|  |  | F (1, 8) = 6.770; P=0.0315 |
|  |  | Drug |
|  |  | F (1, 8) = 10.34; P=0.0123 |
| Figure 2B (85-90 kDa) | two-way ANOVA | Interaction |
|  |  | F (1, 8) = 22.71; P=0.0014 |
|  |  | Genotype |
|  |  | F (1, 8) = 112.0; P<0.0001 |
|  |  | Drug |
|  |  | F (1, 8) = 18.26; P=0.0027 |
| Figure 2C (55-60 kDa) | unpaired t-test, two-tailed | t=1.822, df=6; P=0.1183 |
| Figure 2C (85-90 kDa) | unpaired t-test, two-tailed | t=2.962, df=4; P=0.0415 |
| Figure 2D (55-60 kDa) | unpaired t-test, two-tailed | t=2.899, df=6; P=0.0274 |
| Figure 2D (85-90 kDa) | unpaired t-test, two-tailed | t=5.473, df=6; 0.0016 |
|  | | |
| Figure 3A | One-Way ANOVA | F (DFn, DFd): F (2, 9) = 9,271; P=0.0065 |
| Figure 3B | Unpaired t-test, two-tailed | t, df: t=3,067, df=9; P=0,0134 |
| Figure 3C | Unpaired t-test, two-tailed | t,df: t=2,866, df=9; P=0.0186 |
| Figure 3F (T50) | paired t-test, two-tailed | t, df: t=3.267, df=6; P=0.0171 |
| Figure 3F (T80) | paired t-test, two-tailed | t, df: t=3.540, df=6; P=0.0122 |
|  | | |
| Figure 4A | Two-way ANOVA | interaction |
|  |  | F (1, 28) = 2,053; P=0.1630 |
|  |  | drug |
|  |  | F (1, 28) = 33,85; P<0,0001 |
|  |  | genotype |
|  |  | F (1, 28) = 47,07; P<0,0001 |
| Figure 4B | Two-way ANOVA | interaction |
|  |  | F (1, 40) = 4.540; P=0.0393 |
|  |  | drug |
|  |  | F (1, 40) = 5.854 ; P=0.0202 |
|  |  | genotype |
|  |  | F (1, 40) = 11.40; P=0.0016 |
| Figure 4C | Two-way ANOVA | interaction |
|  |  | F (1, 32) = 0.1920; P=0.6642 |
|  |  | drug |
|  |  | F (1, 32) = 8.449; P=0.0066 |
|  |  | genotype |
|  |  | F (1, 32) = 0.01935; P=0.8902 |
| Figure 4D | Two-way ANOVA | interaction |
|  |  | F (1, 36) = 1.241; P=0.2727 |
|  |  | drug |
|  |  | F (1, 36) = 15.38; P=0.0004 |
|  |  | genotype |
|  |  | F (1, 36) = 1.223; P=0.2761 |
| Figure 4E | Two-way ANOVA | interaction |
|  |  | F (1, 28) = 7.154; P=0.0123 |
|  |  | drug |
|  |  | F (1, 28) = 7.189; P=0.0122 |
|  |  | genotype |
|  |  | F (1, 28) = 17.02; P=0.0003 |
| Figure 4F | Two-way ANOVA | interaction |
|  |  | F (1, 20) = 28.60; P<0.0001 |
|  |  | drug |
|  |  | F (1, 20) = 17.71; P=0.0004 |
|  |  | genotype |
|  |  | F (1, 20) = 8.295; P=0.0093 |
| Figure 4G | Two-way ANOVA | interaction |
|  |  | F (1, 20) = 0.7181; P=0.4068 |
|  |  | drug |
|  |  | F (1, 20) = 0.0002656; P=0.9872 |
|  |  | genotype |
|  |  | F (1, 20) = 0.2681; P=0.6103 |
| Figure 4H | Two-way ANOVA | interaction |
|  |  | F (1, 23) = 0.5646; P=0.4600 |
|  |  | drug |
|  |  | F (1, 23) = 1.448; P=0.2411 |
|  |  | genotype |
|  |  | F (1, 23) = 0.2341; P=0.6331 |
|  | | |
| Figure 5B | Two-way ANOVA | interaction |
|  |  | F (1, 46) = 3.663; P=0.0619 |
|  |  | drug |
|  |  | F (1, 46) = 10.16; P=0.0026 |
|  |  | genotype |
|  |  | F (1, 46) = 7.282; P=0.0097 |
| Figure 5C | Two-way ANOVA | interaction |
|  |  | F (1, 46) = 0.005472; P=0.9414 |
|  |  | drug |
|  |  | F (1, 46) = 1.849; P=0.1805 |
|  |  | genotype |
|  |  | F (1, 46) = 3.839; P=0.0562 |
| Figure 5D | Two-way ANOVA | interaction |
|  |  | F (1, 46) = 3.570; P=0.0651 |
|  |  | drug |
|  |  | F (1, 46) = 8.726; P=0.0049 |
|  |  | genotype |
|  |  | F (1, 46) = 4.199; P=0.0462 |
| Figure 5F | Two-way ANOVA | interaction |
|  |  | F (3, 80) = 12.60; P<0.0001 |
|  |  | Object |
|  |  | F (1, 80) = 37.41; P<0.0001 |
|  |  | group |
|  |  | F (3, 80) = 1.924e-016; P>0.9999 |
| Figure 5G | Two-way ANOVA | interaction |
|  |  | F (1, 40) = 13.40; P=0.0007 |
|  |  | drug |
|  |  | F (1, 40) = 6.460; P=0.0150 |
|  |  | genotype |
|  |  | F (1, 40) = 0.5859; P=0.4485 |
| Figure 5H | Two-way ANOVA | interaction |
|  |  | F (1, 40) = 0.2221; P=0.6400 |
|  |  | drug |
|  |  | F (1, 40) = 0.04525; P=0.8326 |
|  |  | genotype |
|  |  | F (1, 40) = 1.820; P=0.1849 |
| Figure 5I | Two-way ANOVA | interaction |
|  |  | F (1, 40) = 1.003; P=0.3226 |
|  |  | drug |
|  |  | F (1, 40) = 0.4522; P=0.5052 |
|  |  | genotype |
|  |  | F (1, 40) = 0.1058; P=0.7467 |
|  | | |
| Figure 6B | Two-way ANOVA | interaction |
|  |  | F (1, 46) = 9.325; P=0.0038 |
|  |  | drug |
|  |  | F (1, 46) = 0.01129; P=0.9158 |
|  |  | genotype |
|  |  | F (1, 46) = 5.606; P=0.0222 |
| Figure 6C | Two-way ANOVA | interaction |
|  |  | F (1, 45) = 14.30; P=0.0005 |
|  |  | drug |
|  |  | F (1, 45) = 3.553; P=0.0659 |
|  |  | genotype |
|  |  | F (1, 45) = 1.278; P=0.2642 |
| Figure 6D | Two-way ANOVA | interaction |
|  |  | F (1, 46) = 9.571; P=0.0034 |
|  |  | drug |
|  |  | F (1, 46) = 2.382; P=0.1296 |
|  |  | genotype |
|  |  | F (1, 46) = 6.473; P=0.0144 |
| Figure 6E | Two-way ANOVA | interaction |
|  |  | F (1, 46) = 12.54; P=0.0009 |
|  |  | drug |
|  |  | F (1, 46) = 1.029; P=0.3157 |
|  |  | genotype |
|  |  | F (1, 46) = 4.151; P=0.0474 |
| Figure 6G | Two-way ANOVA | interaction |
|  |  | F (1, 56) = 3.917; P=0.0527 |
|  |  | norBNI |
|  |  | F (1, 56) = 10.33; P=0.0022 |
|  |  | Cocaine |
|  |  | F (1, 56) = 9.167; P=0.0037 |
| Figure 6H | Two-way ANOVA | interaction |
|  |  | F (1, 56) = 3.157; P=0.0810 |
|  |  | norBNI |
|  |  | F (1, 56) = 8.994; P=0.0040 |
|  |  | Cocaine |
|  |  | F (1, 56) = 17.81; P<0.0001 |
|  | | |
| Suppl Fig. 1 | One-Way ANOVA | F (DFn, DFd): F (2, 9) = 5.863; P=0.0234 |
|  | | |
| Suppl. Fig. 2 | paired t-test, two-tailed | t, df: t=5.386, df=5; P= 0.0030 |
|  | | |
| Suppl Fig. 3A | Two-way ANOVA | interaction |
|  |  | F (1, 36) = 0.1642; P=0.6877 |
|  |  | drug |
|  |  | F (1, 36) = 14.23; P=0.0006 |
|  |  | genotype |
|  |  | F (1, 36) = 24.06; P<0.0001 |
|  |  |  |
| Suppl Fig. 3B | Two-way ANOVA | interaction |
|  |  | F (1, 28) = 0.1145; P=0.7376 |
|  |  | drug |
|  |  | F (1, 28) = 7.218; P=0.0120 |
|  |  | genotype |
|  |  | F (1, 28) = 1.564; P=0.2215 |
|  | | |
| Suppl. Fig. 4 (saline) | ratio paired t-test, two tailed | t, df: t=3.067, df=6; P= 0.0220 |
| Suppl. Fig. 4 (norBNI) | ratio paired t-test, two tailed | t, df: t=0.6663, df=5; P=0.5347 |
|  | | |
| Suppl. Fig. 5 | unpaired Welch's test, two-tailed | t=0,8486, df=4,177; P=0.4420 |
|  | | |
| Suppl. Fig. 6B | Two-way ANOVA | interaction |
|  |  | F (1, 46) = 8.385; P=0.0058 |
|  |  | drug |
|  |  | F (1, 46) = 4.387; P=0.0418 |
|  |  | genotype |
|  |  | F (1, 46) = 0.2392; P=0.6271 |
| Suppl. Fig. 6C | Two-way ANOVA | interaction |
|  |  | F (1, 46) = 0.2447; P=0.6232 |
|  |  | drug |
|  |  | F (1, 46) = 0.2053; P=0.6526 |
|  |  | genotype |
|  |  | F (1, 46) = 3.554; P=0.0657 |
| Suppl. Fig. 6D | Two-way ANOVA | interaction |
|  |  | F (1, 46) = 7.742; P=0.0078 |
|  |  | drug |
|  |  | F (1, 46) = 1.736; P=0.1942 |
|  |  | genotype |
|  |  | F (1, 46) = 3.835; P=0.0563 |
