# Supplementary figures and images for: Kappa opioid receptor antagonism restores phosphorylation, trafficking and behavior induced by a disease-associated dopamine transporter variant
Source: Mol Psychiatry. 2025 May 29;30(10):4651–64. doi: 10.1038/s41380-025-03055-4 (PMC12436197; doi:10.1038/s41380-025-03055-4)

# NorBNI + U69,593

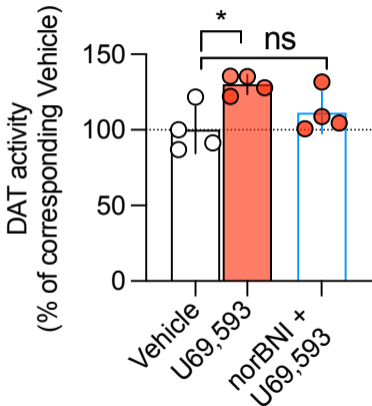

Supplement: Supplementary file 3 — S1 [file 41380_2025_3055_MOESM3_ESM.pdf]

norBNI

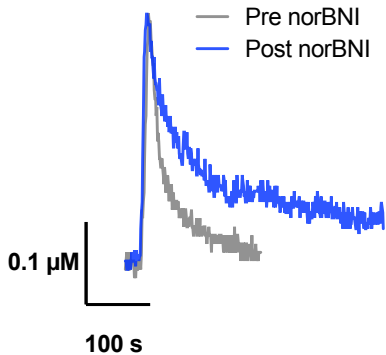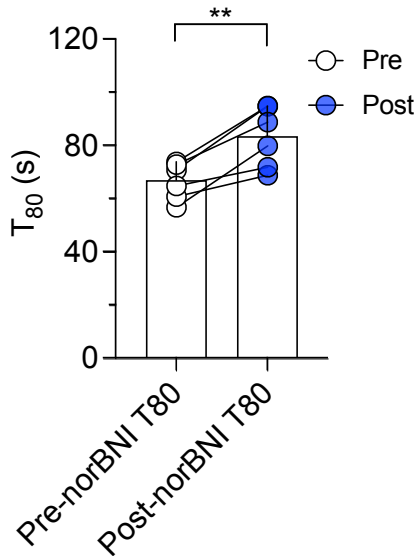

Supplement: Supplementary file 4 — S2 [file 41380_2025_3055_MOESM4_ESM.pdf]

# A Dorsal Striatum

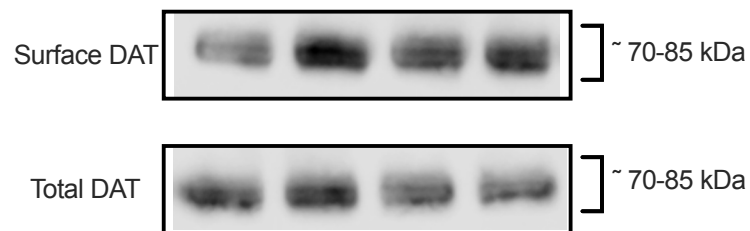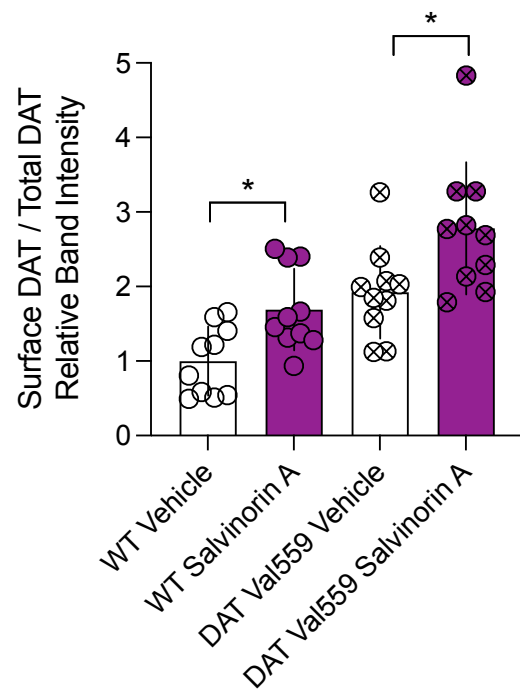

# B Ventral Striatum

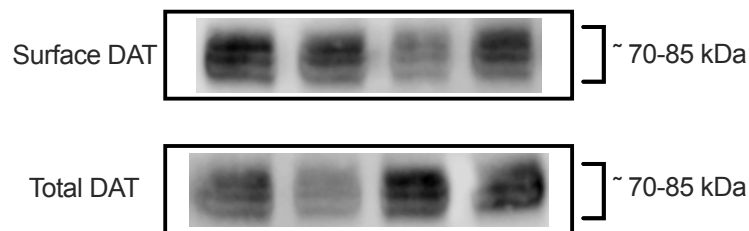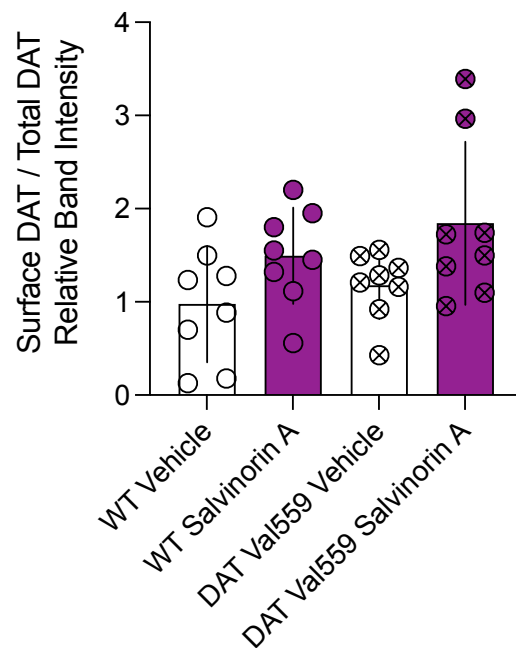

Supplement: Supplementary file 5 — S3 [file 41380_2025_3055_MOESM5_ESM.pdf]

Surface DAT

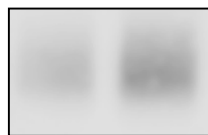

—75 kDa

Total DAT

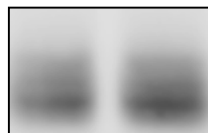

—75 kDa

Saline

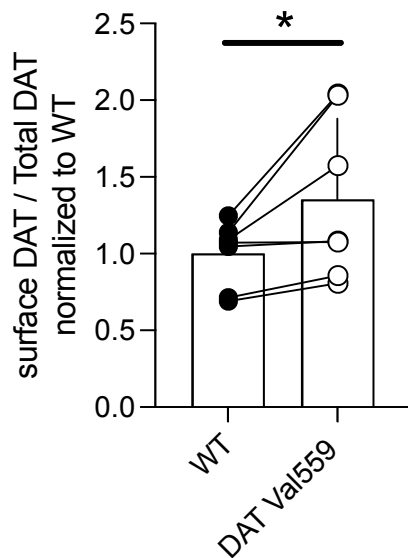

Surface DAT

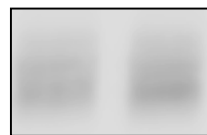

— 75 kDa

Total DAT

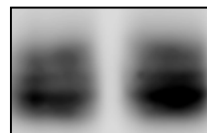

— 75 kDa

norBNI

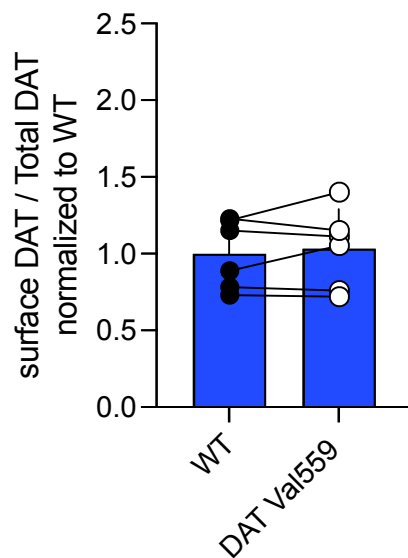

Supplement: Supplementary file 6 — S4 [file 41380_2025_3055_MOESM6_ESM.pdf]

A

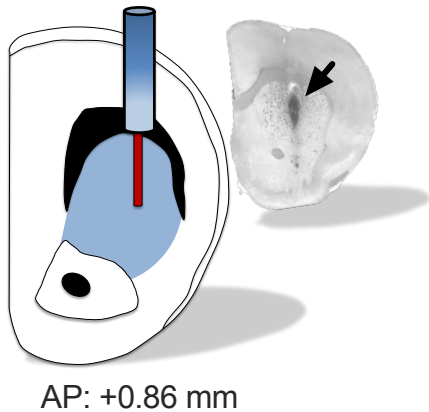

B

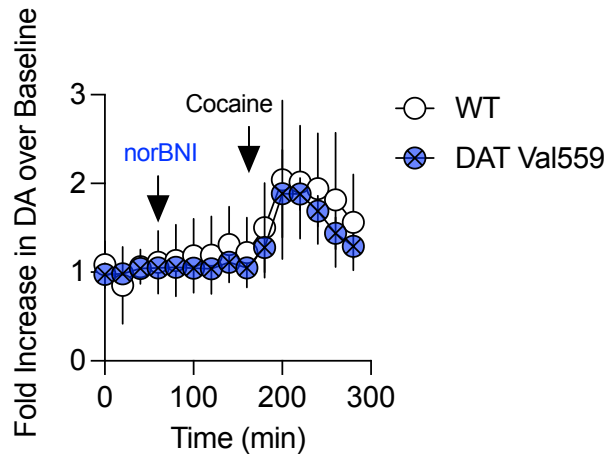

C

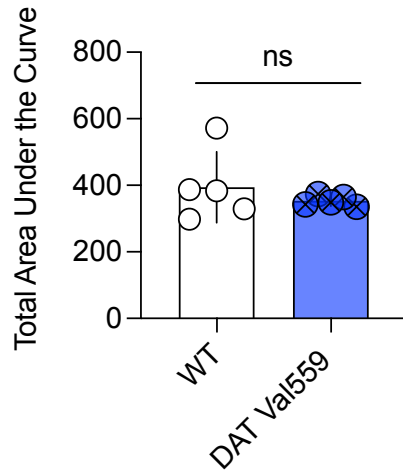

Supplement: Supplementary file 7 — S5 [file 41380_2025_3055_MOESM7_ESM.pdf]

A

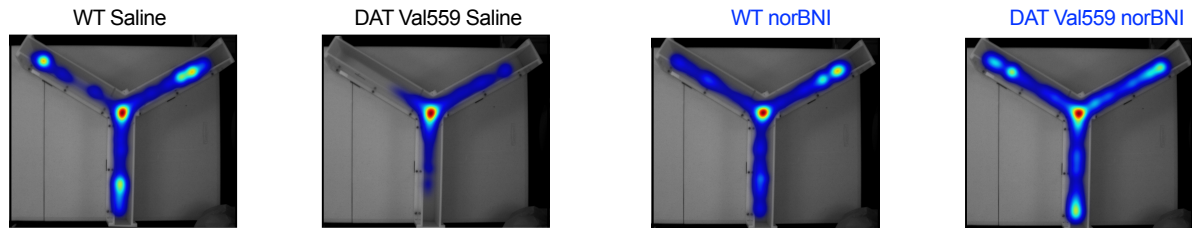

B

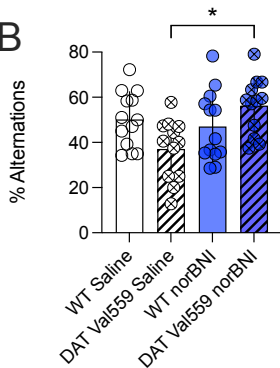

C

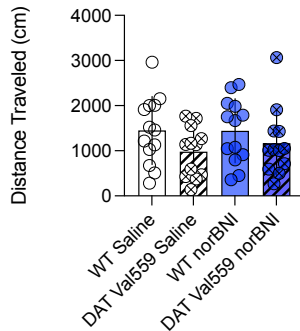

D

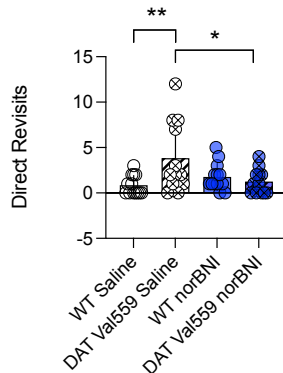

Supplement: Supplementary file 8 — S6 [file 41380_2025_3055_MOESM8_ESM.pdf]
